# Clinical utility of simple subjective gait speed for the risk stratification of heart failure in a primary prevention setting

**DOI:** 10.1038/s41598-022-13752-7

**Published:** 2022-07-08

**Authors:** Kensuke Ueno, Hidehiro Kaneko, Kentaro Kamiya, Hidetaka Itoh, Akira Okada, Yuta Suzuki, Satoshi Matsuoka, Katsuhito Fujiu, Nobuaki Michihata, Taisuke Jo, Norifumi Takeda, Hiroyuki Morita, Junya Ako, Hideo Yasunaga, Issei Komuro

**Affiliations:** 1 The Department of Cardiovascular Medicine, The University of Tokyo Hospital, The University of Tokyo, 7-3-1, Hongo, Bunkyo-ku, Tokyo 113-8655 Japan; 2 Department of Rehabilitation Sciences, Graduate School of Medical Sciences, Kitasato University, Sagamihara, Kanagawa Japan; 3 The Department of Advanced Cardiology, The University of Tokyo, Tokyo, Japan; 4 Department of Rehabilitation, School of Allied Health Sciences, Kitasato University, Sagamihara, Kanagawa Japan; 5 Department of Prevention of Diabetes and Lifestyle-Related Diseases, Graduate School of Medicine, The University of Tokyo, Tokyo, Japan; 6 The Department of Cardiology, New Tokyo Hospital, Matsudo, Japan; 7 The Department of Health Services Research, The University of Tokyo, Tokyo, Japan; 8 Department of Cardiovascular Medicine, Kitasato University School of Medicine, Sagamihara, Kanagawa Japan; 9 The Department of Clinical Epidemiology and Health Economics, School of Public Health, The University of Tokyo, Tokyo, Japan

**Keywords:** Cardiology, Risk factors

## Abstract

Little is known regarding the relationship between self-reported gait speed and the subsequent risk of heart failure (HF) and cardiovascular disease (CVD). We sought to clarify the clinical utility of self-reported gait speed in primary CVD prevention settings. This is an observational cohort study using the JMDC Claims Database, which is an administrative health claims database. Data were collected between January 2005 and April 2020. Medical records of 2,655,359 participants without a prior history of CVD were extracted from the JMDC Claims Database. Gait speed was assessed using information from questionnaires provided at health check-ups, and study participants were categorized into fast or slow gait speed groups. The primary outcome was HF. The secondary outcomes included myocardial infarction (MI), angina pectoris (AP), and stroke. The median age was 45.0 years, and 55.3% of participants were men. 46.1% reported a fast gait speed. The mean follow-up period was 1180 ± 906 days. HF, MI, AP, and stroke occurred in 1.9%, 0.2%, 1.9%, and 1.0% of participants, respectively. Multivariable Cox regression analyses showed that, compared with slow gait speed, fast gait speed was associated with a lower incidence of HF, MI, AP, and stroke. The discriminative predictive ability for HF significantly improved by adding self-reported gait speeds to traditional risk factors (net reclassification improvement 0.0347, *p* < 0.001). In conclusion, our analysis demonstrated that subjective gait speed could be a simple method to stratify the risk of HF and other CVD events in the general population. Further investigations are required to clarify the underlying mechanism of our results and to develop a novel approach for primary CVD prevention.

## Introduction

Gait speed is clinically utilized as an indicator of physical performance and functional capacity of patients with various cardiovascular diseases (CVDs) because it denotes the health and functional status of CVD patients^1–5^. Objectively measured gait speed was reported to be associated with the clinical outcomes of patients with CVD^3,5–10^. For example, gait speed measured objectively using a 4-m walkway was reported to be associated with all-cause mortality and risk for heart failure (HF) admission in elderly HF patients^8^. Similarly, gait speed measured using a 5-m walkway contributed to the identification of vulnerable patients at incrementally higher risk of mortality and major morbidity after cardiac surgery in elderly patients^6^. Therefore, gait speed is recognized as the sixth vital sign^1,4,11^, and objective gait speed measurement is mainly used for risk stratification in secondary CVD prevention settings. On the other hand, there are several issues to be clarified regarding the use of gait speed in primary CVD prevention. First, objective gait speed measurement as a screening tool for the general population in primary CVD prevention is not feasible because it necessitates measurement with attendance involving a large number of patients and medical staff, and thus is time-consuming and laborious. Given this situation, subjective gait speed measurements may be an acceptable option. Second, subjective gait speed is known to reflect functional capacity as well as objective gait speed^12,13^. However, there are limited data on the association between subjective gait speed and incident CVD in the general population. Third, clinical evidence regarding gait speed and CVD comes mostly from data obtained in elderly populations or those with CVD history^5–8,10,14–16^, and there has been no epidemiological data regarding the relationship between gait speed (particularly subjective gait speed) and incident CVD among young or middle-aged people who are the primary targets for primary CVD prevention. Accordingly, we analyzed a large-scale population-based dataset including primarily working age people and aimed to examine the usefulness of subjective gait speed as a screening tool for subsequent CVD occurrence in those without CVD. In particular, we focused on the association of subjective gait speed with incident HF, which is becoming increasingly recognized for its clinical importance, in the present study. Furthermore, we aimed to evaluate the predictive value of subjective gait speed for HF and CVD events in addition to conventional CVD risk factors.

## Materials and methods

This database is available for anyone who purchases it from JMDC Inc. (https://www.jmdc.co.jp/en/index).

### Study design and data source

This study is a retrospective observational analysis using data from the JMDC Claims Database (JMDC; Tokyo, Japan), a health check-up and claims database, collected between January 2005 and April 2020^17,18^. The JMDC contracts with more than 60 insurers and includes data for health insurance records of registered individuals. Most individuals registered in the JMDC Claims Database are employees of relatively large Japanese companies. Detailed information on this database is described in Yasunaga et al.^19^. This dataset contains the annual health check-up data, including questionnaires regarding gait speed. Data on clinical follow-ups obtained from administrative claims records are also included in this dataset. Incidences of CVD, including HF, myocardial infarction (MI), angina pectoris (AP), and stroke, were evaluated using the International Classification of Disease, 10th Revision (ICD-10) diagnosis codes recorded in the claim records of each individual^20^.

**Figure 1:**
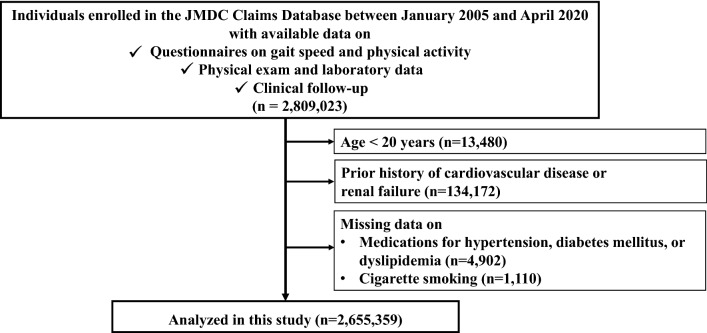
We extracted the data of 2,809,023 individuals who were enrolled in the JMDC Claims Database between January 2005 and April 2020 and whose baseline health check-up data (including data on gait speed) were available. Exclusion criteria were as follows: (1) age < 20 years (n = 13,480); (2) prior history of heart failure, myocardial infarction, angina pectoris, stroke, or renal failure (n = 134,172); and (3) missing data on medications for hypertension, diabetes mellitus, or dyslipidemia (n = 4902) and cigarette smoking (n = 1110). Finally, we analyzed 2,655,359 participants in this study.

We extracted the data of 2,809,023 individuals enrolled in the JMDC Claims Database between January 2005 and April 2020 whose baseline health check-up data (including gait speed) were available. Exclusion criteria were as follows: (1) age < 20 years (n = 13,480); (2) prior history of HF, MI, AP, stroke, or renal failure (n = 134,172); and (3) missing data on medications for hypertension, diabetes mellitus, or dyslipidemia (n = 4,902) and cigarette smoking (n = 1110). Following these criteria, we analyzed the 2,655,359 qualified participants in this study (Fig. 1).

### Ethics

This study was conducted according to the ethics guidelines of our institution (approval by the Institutional Review Board of the University of Tokyo: 2018-10862) in accordance with the principles of the Declaration of Helsinki. The requirement for informed consent was waived because all individuals in this dataset were de-identified. All data were compliant with the International Conference on Harmonization guidelines^21^.

### Gait speed

We obtained information regarding gait speed from the questionnaires in the health check-up records. The questionnaires are nearly uniform because for most Japanese employees a mandatory regular health check-up is conducted under the jurisdiction of the Ministry of Health, Labour, and Welfare using a standardized protocol. If a study participant answered “YES” to the following question: “Do you walk faster than others of the same age and sex?”, the study participants were categorized as having fast gait speed. If a study participant answered “NO” to this question, then this study participant was categorized as having slow gait speed.

### Definition

Obesity was defined as a body mass index ≥ 25 kg/m^2^. Hypertension was defined as systolic blood pressure ≥ 140 mmHg, diastolic blood pressure ≥ 90 mmHg, or use of antihypertensive medications. Diabetes mellitus was defined as a fasting glucose level ≥ 126 mg/dL or the use of antidiabetic medications including insulin. Dyslipidemia was defined as low-density lipoprotein cholesterol level ≥ 140 mg/dL, high-density lipoprotein cholesterol level < 40 mg/dL, triglyceride level ≥ 150 mg/dL, or use of lipid-lowering medications. Information on cigarette smoking (current or non-current) and physical activity was self-reported. Physical inactivity was defined as not engaging in 30 min of exercise at least twice a week or not walking ≥ 1 h per day^17^.

### Clinical outcomes

Clinical follow-up was initiated from the date of the initial health check-up for each participant, and outcome data were collected between January 2005 and April 2020. We defined the primary outcome as HF (ICD-10 codes: I500, I501, I509, and I110). We also defined secondary outcomes as MI (ICD-10 codes: I210–I214 and I219), AP (ICD-10 codes: I200, I201, I208, and I209), and stroke (ICD-10 codes: I630, I631–I636, I638, I639, I600–I611, I613–I616, I619, I629, and G459). Each CVD event was separately analyzed. For example, if a participant experienced stroke and then MI seven months later, we counted both stroke and MI events as separate outcomes.

### Statistical procedures

Categorical and continuous data of the baseline characteristics are presented as percentages (%) and medians (with interquartile range [IQR]). A chi-square test was used to compare categorical variables between participants with fast and slow gait speeds. Unpaired t-tests were used to compare continuous variables between the two groups. Cox regression analysis was used to uncover the relationship between fast gait speed and the incidence of each CVD event. Model 1 included fast gait speed alone (unadjusted model); model 2 included the hazard ratios (HRs) of fast gait speed adjusted for age and sex, and model 3 included the HRs of fast gait speed adjusted for age, sex, obesity, hypertension, diabetes mellitus, dyslipidemia, cigarette smoking, and physical inactivity. Four sensitivity analyses were performed. First, to account for the missing data, we also conducted multiple imputations as previously described^22,23^. On the assumption of data missing at random, the missing data was imputed for covariates using the chained equation method with 20 iterations as described by Aloisio et al.^24^ HRs and standard errors were calculated using Rubin’s rules^25^. Second, as subgroup analyses, our study population was divided by age (≥ 50 years, < 50 years), sex (men, women), obesity (obese, non-obese), hypertension, diabetes mellitus, dyslipidemia, and physical activity. Multivariable Cox regression analyses were conducted in each subgroup. *p* values for multiplicative interactions between subgroups were then calculated. Third, 2,175,267 participants whose follow-up period was ≥ 365 days were analyzed. Fourth, we adjusted our primary results for a prior history of peripheral artery disease defined as ICD-10 codes of I702 and I709.

Net reclassification improvement (NRI) analysis was performed to calculate the discrimination predictive value of gait speed for CVD events^26^. The results were considered statistically significant at *p* < 0.05. All statistical analyses were performed using SPSS software (version 25, IBM Corp, Armonk, NY, USA) and Stata software (version 17; StataCorp LLC, College Station, TX, USA).

## Results

### Clinical characteristics

Characteristics of the study participants are shown in Table 1. Overall, the median age was 45.0 (IQR, 38.0–53.0) years, and 1,468,347 participants (55.3%) were men. Among the total cohort, 1,223,871 participants (46.1%) reported having a fast gait speed. Obesity, hypertension, diabetes mellitus, and physical inactivity were less common in participants with fast gait speed than in those with slow gait speed.

**Table 1:** Clinical Characteristics.

Variable	Slow gait speed (n = 1,431,488)	Fast gait speed (n = 1,223,871)	*p* value
Age (years)	44.0 (38.0–52.0)	46.0 (39.0–54.0)	< 0.001
Men	741,022 (51.8%)	727,325 (59.4%)	< 0.001
Body mass index (kg/m^2^)	22.4 (20.2–25.1)	22.2 (20.2–24.5)	< 0.001
Obesity	368,811 (25.8%)	262,063 (21.4%)	< 0.001
Hypertension	253,359 (17.7%)	214,372 (17.5%)	< 0.001
Systolic blood pressure (mmHg)	117.0 (107.0–128.0)	118.0 (107.0–128.0)	< 0.001
Diastolic blood pressure (mmHg)	72.0 (64.0–81.0)	73.0 (65.0–81.0)	< 0.001
Diabetes mellitus	64,058 (4.5%)	46,979 (3.8%)	< 0.001
Dyslipidemia	553,619 (38.7%)	476,920 (39.0%)	< 0.001
Cigarette smoking	351,076 (24.5%)	317,945 (26.0%)	< 0.001
Physical inactivity	871,553 (60.9%)	526,708 (43.0%)	< 0.001
**Laboratory data**			
Glucose (mg/dL)	91.0 (85.0–98.0)	92.0 (86.0–99.0)	0.010
Low-density lipoprotein cholesterol (mg/dL)	118.0 (98.0–140.0)	118.0 (98.0–140.0)	< 0.001
High-density lipoprotein cholesterol (mg/dL)	62.0 (52.0–74.0)	63.0 (52.0–75.0)	< 0.001
Triglycerides (g/dL)	80.0 (56.0–121.0)	80.0 (57.0–120.0)	0.017

Data are expressed as median (interquartile range) or number (percentage). We obtained information on gait speed from questionnaires during health check-ups. If a study participant answered “YES” to the following question: “Do you walk faster than others of the same age and sex?” then this study participant was categorized as having fast gait speed. If a study participant answered “NO” to this question, then this study participant was categorized as having slow gait speed.

### Gait speed and heart failure events

**Figure 2:**
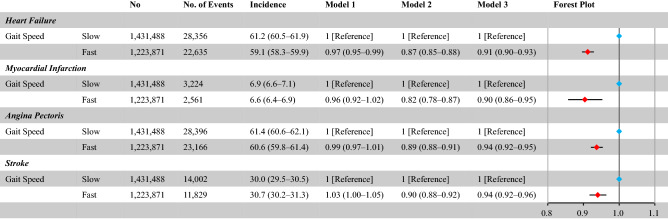
The frequency of events, corresponding incidence rates, and hazard ratios of fast gait speed for cardiovascular disease events. The incidence rate was per 10,000 person-years. Cox regression analyses; Model 1 included fast gait speed alone (unadjusted model); model 2 included the hazard ratios (HRs) of fast gait speed adjusted for age and sex, and model 3 included the HRs of fast gait speed adjusted for age, sex, obesity, hypertension, diabetes mellitus, dyslipidemia, cigarette smoking, and physical inactivity.

During a mean follow-up of 1180 ± 906 days, 50,991 (1.9%) HF events occurred. The incidence of HF was 59.1 (95% confidence interval (CI), 58.3–59.9) per 10,000 person-years in participants with fast gait speed and 61.2 (95% CI, 60.5–61.9) per 10,000 person-years in participants with slow gait speed. Univariate and age-sex-adjusted Cox regression analyses showed that fast gain speed was associated with a lower incidence of HF. Further, multivariable Cox regression analysis demonstrated that fast gait speed was associated with a lower risk of HF incidence (HR 0.91, 95% CI 0.90–0.93) (Fig. 2).

### Gait speed and other cardiovascular events

During the follow-up period, MI, AP, and stroke occurred in 5785 (0.2%), 51,562 (1.9%), and 25,831 (1.0%) patients, respectively. Multivariable Cox regression analyses showed that fast gait speed was associated with a lower incidence of MI (HR 0.90, 95% CI 0.86–0.95), AP (HR 0.94, 95% CI 0.92–0.95), and stroke (HR 0.94, 95% CI 0.92–0.96) (Fig. 2).

### Sensitivity analyses

**Figure 3:**
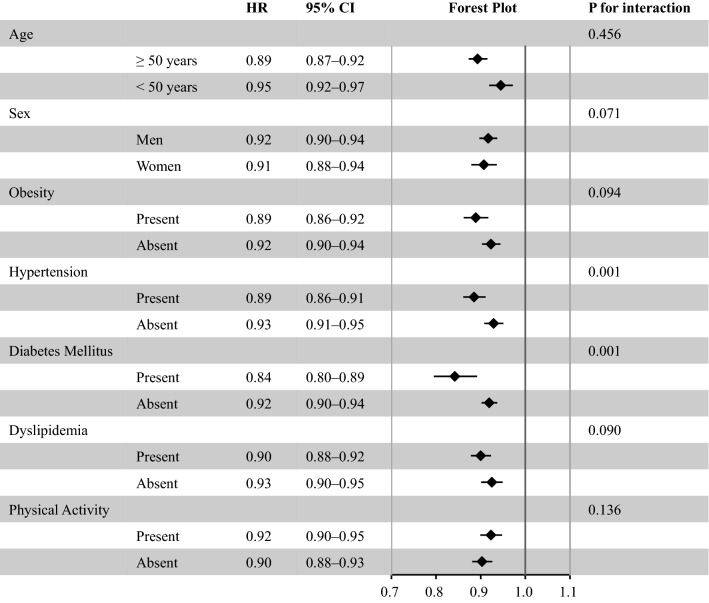
Hazard ratios of fast gait speed for the risk of heart failure in each subgroup. Adjusted with age, sex, obesity, hypertension, diabetes mellitus, dyslipidemia, cigarette smoking, and physical inactivity in the subgroup analyses stratified by age. Adjusted for age, obesity, hypertension, diabetes mellitus, dyslipidemia, cigarette smoking, and physical inactivity in the subgroup analyses stratified by sex. Adjusted with age, sex, hypertension, diabetes mellitus, dyslipidemia, cigarette smoking, and physical inactivity in the subgroup analyses stratified by the presence of obesity. Adjusted with age, sex, obesity, diabetes mellitus, dyslipidemia, cigarette smoking, and physical inactivity in the subgroup analyses stratified by hypertension. Adjusted with age, sex, obesity, hypertension, dyslipidemia, cigarette smoking, and physical inactivity in the subgroup analyses stratified by diabetes mellitus. Adjusted with age, sex, obesity, hypertension, diabetes mellitus, cigarette smoking, and physical inactivity in the subgroup analyses stratified by dyslipidemia. Adjusted with age, sex, obesity, hypertension, diabetes mellitus, dyslipidemia, and cigarette smoking in the subgroup analyses stratified by the presence of physical activity. *HR* Hazard ratio, *CI* Confidence interval.

We performed four sensitivity analyses. After imputing missing data, 2,661,371 participants were analyzed with a mean follow-up of 1179 ± 906 days. During this follow-up period, 51,059 HF, 5789 MI, 51,624 AP, and 25,869 stroke events were recorded. Multivariable Cox regression analyses also showed that fast gait speed was associated with a lower incidence of HF (HR 0.91, 95% CI 0.90–0.93), MI (HR 0.91, 95% CI 0.86–0.95), AP (HR 0.94, 95% CI 0.92–0.95), and stroke (HR 0.94, 95% CI 0.92–0.96), as shown in Supplementary Table 1. The results of the subgroup analyses are summarized in Fig. 3. The association between fast gait speed and the risk of HF incidence was consistent in each subgroup. Fast gait speed was associated with a lower incidence of HF (HR 0.91, 95% CI 0.89–0.93) even in participants whose follow-up period for HF was longer than one year (Supplementary Table 2). Our main results were unchanged after adjustment for a prior history of peripheral artery disease (Supplementary Table 3).

### Net reclassification improvement

When self-reported gait speed was added to age, sex, obesity, hypertension, diabetes mellitus, dyslipidemia, cigarette smoking, and physical inactivity, the NRI for HF, MI, AP, and stroke was 0.0347 (95% CI 0.0259–0.0434, *p* < 0.001), 0.0365 (95% CI 0.0107–0.0623, *p* = 0.0055), 0.0237 (95% CI 0.0150–0.0324, *p* < 0.001), and 0.0060 (95% CI − 0.0063 to + 0.0183, *p* = 0.3372), respectively.

## Discussion

This comprehensive analysis of a nationwide epidemiological database included approximately 2.6 million individuals with no prior history of CVD. We found that fast gait speed was independently associated with a lower risk of incident HF, MI, AP, and stroke. Furthermore, adding gait speed to traditional risk factors may help improve the predictive ability for HF events. To the best of our knowledge, this is the first clinical evidence using a large-scale epidemiological dataset demonstrating the relationship between self-reported gait speed and incident HF and CVD in the general population. This highlights a potential clinical utility as a simple predictive indicator of subjective gait speed in primary CVD prevention.

In the field of secondary CVD prevention, various studies have confirmed the prognostic utility of gait speed (mainly, objective gait speed measurement) for CVD outcomes^3,5–10^. However, data on the association of gait speed with incident CVD in primary prevention settings are scarce. The present study is distinguishable from previous studies in that we demonstrated the potential utility of subjective gait speed for risk stratification in primary CVD prevention. We found that gait speed, which was obtained using a self-report questionnaire, was associated with incident HF and other CVD events in the general population. Further, subgroup analyses showed that this association was present irrespective of age, sex, obesity, dyslipidemia, and physical activity at baseline.

Several possible mechanisms could explain the relationship between gait speed and subsequent risk of HF and other CVD events. First, slow gait speed may reflect the presence of other comorbidities and physical inactivity^27,28^. However, even after adjustment for various covariates, including physical inactivity, the relationship between gait speed and incident HF was present in this study, and there were no interactions of sex (men and women), age (≥ 50 years and < 50 years), obesity (obese and non-obese), dyslipidemia, or physical activity (active and inactive). Second, slow gait speed is commonly associated with enhanced inflammation and oxidative stress^29^, which could contribute to the development of HF and CVD^30^. Third, self-reported gait speed has been reported to be associated with not only objectively measured gait speed, but also physical functions reflecting skeletal muscle function, such as grip strength, leg strength, Short Physical Performance Battery, and 6-min walking distance^12,13^. Skeletal muscle mass and muscle strength are the main determinants of gait speed^31–34^. Decreased skeletal muscle mass leads to decreased cardiac function due to increased ergoreflex^35,36^. Any of these factors represent potential mechanisms that could explain the results of this study. Further investigations are required to uncover the link between gait speed and the subsequent risk of developing HF and other CVD events.

This study has several clinical implications. CVD is the leading cause of morbidity and mortality in developed countries^37–39^, and primary CVD prevention is an essential factor in reducing the CVD burden. For this purpose, a simple and appropriate risk stratification is warranted for primary CVD prevention. In particular, HF is still increasing in prevalence, and therefore, estimating an individual’s risk of subsequent HF development in the general population is important. Objective gait speed measurement is an established indicator for risk stratification of patients with CVD in secondary CVD prevention settings. However, considering the feasibility in primary prevention for the general population, subjective gait speed assessment represents a better option because self-reported gait speed can easily be obtained using a questionnaire. Self-reported gait speed assessment takes only a few seconds and does not necessitate any laborious procedures or special facilities. Therefore, we believe that subjective gait speed assessment is feasible in a real-world clinical setting and would provide information on the risk of incident HF and CVD.

This study has several limitations. We performed multivariable Cox regression analyses. However, we were unable to eliminate the potential impacts of unmeasured confounders and residual bias. Since the population included in this dataset mainly comprised employed, working-aged people, we acknowledge a selection bias (skewing toward healthy workers), which might limit the generalizability of our findings. Slow gait speed itself could be a sign of latent HF; however, when we analyzed participants with a follow-up period for HF of longer than one year, our primary results did not change. Detailed data on cardiac function (e.g., brain natriuretic peptide level, echocardiographic parameter) were unavailable in our dataset. Although lifestyle modifications or changes may have been undertaken after the health check-up, which could have affected our results, these factors were not considered in the present study.

In conclusion, our comprehensive analyses of a nationwide population-based database demonstrated that subjectively fast gait speed was associated with a lower risk of incident HF and a variety of CVD events among the general population. This suggests potential clinical utility of subjective gait speed assessment in primary CVD prevention settings. Furthermore, the evaluation of gait speed may have the potential to improve predictive ability for future HF and CVD events. This demonstrates the essential role of functional exercise ability in primary CVD prevention.

### Transparency declaration

The manuscript’s guarantor (HK) affirms that the manuscript is an honest, accurate, and transparent account of the study being reported; that no important aspects of the study have been omitted; and that any discrepancies from the study as originally planned have been explained.

## Supplementary Information

Supplementary Information.

## Data Availability

The JMDC Claims Database is not publicly available due to contracts with the JMDC, which is a medical venture company. However, the JMDC Claims Database is available for purchase from JMDC Inc. (https://www.jmdc.co.jp/en/index). Stata codes used for the statistical procedures in this study are available from the corresponding author on reasonable request. The requirement for informed consent was waived because all data from the JMDC Claims Database were deidentified.
