# Supplementary material for: Clinical utility of simple subjective gait speed for the risk stratification of heart failure in a primary prevention setting
Source: Sci Rep. 2022 Jul 8;12:11641. doi: 10.1038/s41598-022-13752-7 (PMC9270451; doi:10.1038/s41598-022-13752-7)
Supplement: Supplementary file 1 — Supplementary Information. [file 41598_2022_13752_MOESM1_ESM.docx]

| **Supplementary Table 1. Multivariable Cox regression analysis for cardiovascular events after multiple imputations.** | | |
| --- | --- | --- |
|  | Slow Gait Speed  (n=1,434,614) | Fast Gait Speed  (n=1,226,757) |
| Heart Failure |  |  |
| No. of Events | 28,388 | 22,671 |
| Incidence (per 10,000 person-years) | 61.1 (60.4–61.9) | 59.1 (58.3–59.9) |
| Model 1 | 1 [Reference] | 0.97 (0.95­–0.99) |
| Model 2 | 1 [Reference] | 0.87 (0.85–0.88) |
| Model 3 | 1 [Reference] | 0.91 (0.90–0.93) |
| Myocardial Infarction |  |  |
| No. of Events | 3,225 | 2,564 |
| Incidence (per 10,000 person-years) | 6.9 (6.6–7.1) | 6.6 (6.4–6.9) |
| Model 1 | 1 [Reference] | 0.97 (0.92–1.02) |
| Model 2 | 1 [Reference] | 0.83 (0.78–0.87) |
| Model 3 | 1 [Reference] | 0.91 (0.86–0.95) |
| Angina Pectoris |  |  |
| No. of Events | 28,430 | 23,194 |
| Incidence (per 10,000 person-years) | 61.3 (60.6–62.1) | 60.6 (59.8–61.4) |
| Model 1 | 1 [Reference] | 0.99 (0.97–1.00) |
| Model 2 | 1 [Reference] | 0.89 (0.88–0.91) |
| Model 3 | 1 [Reference] | 0.94 (0.92–0.95) |
| Stroke |  |  |
| No. of Events | 14,023 | 11,846 |
| Incidence (per 10,000 person-years) | 30.0 (29.5–30.5) | 30.7 (30.2–31.3) |
| Model 1 | 1 [Reference] | 1.03 (1.00–1.05) |
| Model 2 | 1 [Reference] | 0.90 (0.88–0.92) |
| Model 3 | 1 [Reference] | 0.94 (0.92–0.96) |

We analyzed 2,661,371 participants after multiple imputations for missing data. The incidence rate was per 10,000 person-years. Cox regression analyses; Model 1 included fast gait speed alone (unadjusted model); model 2 included the hazard ratios (HRs) of fast gait speed adjusted for age and sex, and model 3 included the HRs of fast gait speed adjusted for age, sex, obesity, hypertension, diabetes mellitus, dyslipidemia, cigarette smoking, and physical inactivity.

| **Supplementary Table 2. Multivariable Cox regression analysis for cardiovascular events in participants whose follow-up for HF ≥365 days** | | |
| --- | --- | --- |
|  | Slow Gait Speed  (n=1,176,216) | Fast Gait Speed  (n=999,051) |
| Heart Failure |  |  |
| No. of Events | 21,109 | 16,705 |
| Incidence (per 10,000 person-years) | 63.5 (62.6–64.4) | 61.6 (60.6–62.5) |
| Model 1 | 1 [Reference] | 0.97 (0.95–0.99) |
| Model 2 | 1 [Reference] | 0.86 (0.85–0.88) |
| Model 3 | 1 [Reference] | 0.91 (0.89–0.93) |

We analyzed 2,175,267 participants whose follow-up period for HF ≥365 days. The incidence rate was per 10,000 person-years. Cox regression analyses; Model 1 included fast gait speed alone (unadjusted model); model 2 included the hazard ratios (HRs) of fast gait speed adjusted for age and sex, and model 3 included the HRs of fast gait speed adjusted for age, sex, obesity, hypertension, diabetes mellitus, dyslipidemia, cigarette smoking, and physical inactivity.

| **Supplementary Table 3. Multivariable Cox regression analysis for cardiovascular events.** | | |
| --- | --- | --- |
|  | Slow Gait Speed  (n=1,431,488) | Fast Gait Speed  (n=1,223,871) |
| Heart Failure |  |  |
| History of peripheral artery disease | 1 [Reference] | 0.97 (0.95–0.99) |
| Model 2 and history of peripheral artery disease | 1 [Reference] | 0.87 (0.85–0.88) |
| Model 3 and history of peripheral artery disease | 1 [Reference] | 0.91 (0.90–0.93) |
| Myocardial Infarction |  |  |
| History of peripheral artery disease | 1 [Reference] | 0.96 (0.92–1.02) |
| Model 2 and history of peripheral artery disease | 1 [Reference] | 0.82 (0.78–0.87) |
| Model 3 and history of peripheral artery disease | 1 [Reference] | 0.90 (0.86–0.95) |
| Angina Pectoris |  |  |
| History of peripheral artery disease | 1 [Reference] | 0.99 (0.97–1.01) |
| Model 2 and history of peripheral artery disease | 1 [Reference] | 0.89 (0.88–0.91) |
| Model 3 and history of peripheral artery disease | 1 [Reference] | 0.94 (0.92–0.95) |
| Stroke |  |  |
| History of peripheral artery disease | 1 [Reference] | 1.03 (1.00–1.05) |
| Model 2 and history of peripheral artery disease | 1 [Reference] | 0.90 (0.88–0.92) |
| Model 3 and history of peripheral artery disease | 1 [Reference] | 0.94 (0.92–0.96) |

We examined whether a history of peripheral artery disease would affect the primary results.

Cox regression analyses; Model 2 included the hazard ratios (HRs) of fast gait speed adjusted for age and sex, and model 3 included the HRs of fast gait speed adjusted for age, sex, obesity, hypertension, diabetes mellitus, dyslipidemia, cigarette smoking, and physical inactivity.
